# Development and Validation of a LC–MS/MS-Based Assay for Quantification of Free and Total Omega 3 and 6 Fatty Acids from Human Plasma

**DOI:** 10.3390/molecules24020360

**Published:** 2019-01-20

**Authors:** Vlad Serafim, Diana-Andreea Tiugan, Nicoleta Andreescu, Alexandra Mihailescu, Corina Paul, Iulian Velea, Maria Puiu, Mihai Dinu Niculescu

**Affiliations:** 1 Genetics Discipline, Centre of Genomic Medicine Timișoara, “Victor Babeș” University of Medicine and Pharmacy, No 2, Eftimie Murgu Square, Timișoara 300041, Romania; vladserafim@gmail.com (V.S.); tiugandiana@gmail.com (D.-A.T.); alex.fifu@gmail.com (A.M.); maria_puiu@umft.ro (M.P.); mihai.niculescu@gmail.com (M.D.N.); 2 “Louis Țurcanu” Clinical Emergency Hospital for Children, No 2, Iosif Nemoianu St., Timișoara 300011, Romania; 3 Pediatric Department, “Victor Babeș” University of Medicine and Pharmacy, No 2, Eftimie Murgu Square, Timișoara 300041, Romania; corinapaul17@gmail.com (C.P.); ivelea56@yahoo.com (I.V.)

**Keywords:** high-performance liquid chromatography, tandem mass spectrometry, polyunsaturated fatty acids, metabolomics, negative electrospray ionization, human plasma, C18 column, PUFA, MRM

## Abstract

Few high-performance liquid chromatography–tandem mass spectrometry (LC-MS/MS) methods have been developed for the full quantitation of fatty acids from human plasma without derivatization. Therefore, we propose a method that requires fewer sample preparation steps, which can be used for the quantitation of several polyunsaturated fatty acids in human plasma. The method offers rapid, accurate, sensitive, and simultaneous quantification of omega 3 (α-linolenic, eicosapentaenoic, and docosahexaenoic acids) and omega 6 fatty acids (arachidonic and linoleic acids) using high-performance LC-MS/MS. The selected fatty acids were analysed in lipid extracts from both free and total forms. Chromatographic separation was achieved using a reversed phase C18 column with isocratic flow using ammonium acetate for improving negative electrospray ionization (ESI) response. Mass detection was performed in multiple reaction monitoring (MRM) mode, and deuterated internal standards were used for each target compound. The limits of quantification were situated in the low nanomolar range, excepting linoleic acid, for which the limit was in the high nanomolar range. The method was validated according to the U.S. Department of Health and Human Services guidelines, and offers a fast, sensitive, and reliable quantification of selected omega 3 and 6 fatty acids in human plasma.

## 1. Introduction

Polyunsaturated fatty acids (PUFAs) play essential roles in human physiology, and are both obtained from foods and synthesized endogenously. Dietary habits have changed in the last decades, especially in the western world where the intake ratio between polyunsaturated omega 3 (*n*-3) and omega 6 (*n*-6) fatty acids has shifted from 1:1 to 20:1 in favour of *n*-3. Studies revealed that this modified ratio has several health implications, influencing health outcomes through several mechanisms such as systemic inflammation, adipogenesis, browning of adipose tissue, lipid homeostasis, and the brain–gut–adipose-tissue communication [1]. Dietary supplementation with *n*-3 fatty acids has proven effective in reducing the risk of heart disease, triglycerides levels, symptoms of rheumatoid arthritis, and progression of eye diseases [2,3,4]. As such, supplementation with PUFAs enhanced the expression of genes involved in insulin signalling and glucose transport [5].

The *n*-3 fatty acids family includes α-linolenic acid (ALA), eicosapentaenoic acid (EPA), and docosahexaenoic acid (DHA). ALA, one of the two essential fatty acids (FAs) along with linoleic acid, is available mostly from plant seeds (chia, flaxseed, hemp) and from other plant oils [6]. DHA and EPA (also *n*-3 FAs) can be synthetized from ALA through desaturation and elongation, but with relative low efficiency [7]. The main dietary sources of DHA and ALA are marine algae and fish oil. DHA is a major constituent of brain, skin, and retina, while EPA is a precursor for eicosanoids [6]. The other essential FAs, linoleic acid (LA), is a *n*-6 FA available from vegetable oils, seeds, nuts, eggs, and meat and, apart from its role as an energy source, it is a constituent of cell membranes, and its derivate molecules are involved in cell signalling. LA is converted into gamma linolenic (GLA) by a Δ^6^ desaturase enzyme. GLA is further converted into dihomo gamma linolenic (DGLA), conversion which is enhanced by high levels of ALA. DGLA is converted into arachidonic acid (ARA), a *n*-6 FA that is an important constituent of phospholipids in cell membranes, and is also involved in cell signalling [8].

Considering their important physiological roles in an organism, accurate plasma quantification of *n*-3 and *n*-6 FAs (from both their free and total forms) is used in many studies.

Until recently, FAs were commonly quantified using gas chromatography–mass spectrometry (GS-MS) with electron impact ionization following methyl esters derivatization [9,10] and gas chromatography-flame ionization (GS-FID) [11]. Liquid chromatography–mass spectrometry (LC–MS) began more recently to be used for FAs analysis. Using electrospray ionization (ESI), FAs tend to ionize in negative mode, but they were reported to exhibit low specificity [12]. Therefore, chemical derivatization was used to improve the ESI-LC-MS detection [12,13,14]. However, derivatization requires additional steps in the sample preparation protocol. Several LC-MS methods for analysing underivatized FAs were published, but these methods did not use tandem MS detection [15,16], or they were focused on qualitative analysis [17]. Underivatized fatty acids were also quantitatively analysed with barium acetate as the cationization agent in the positive ionization mode with a limit of detection in low micromolar range [18]. Additionally, only few reports focused on quantifying both free and total FAs together.

The aim of this study was to develop and validate a LC-MS/MS method to fully quantitate ALA, ARA, DHA, EPA, and LA in plasma from both their free and total forms, using a simplified sample preparation procedure without the need of chemical derivatization. The method is intended as an improved alternative to the existing gas chromatography-based methods by offering shorter analytical runs.

## 2. Results and Discussion

### 2.1. Comparison of the Two Extraction Procedures

The lipid extraction method with hexane/isopropanol was adapted after a method described by Hara and Radin [19] for extracting lipids from nervous tissue. By comparing it to the Bligh–Dyer method, we found that the two methods gave similar results for free FAs extraction. However, for total FAs extraction, the hexane/isopropanol method proved to be more efficient as most FAs levels were higher (see Figure S2).

A previous study by Reis et al. [20] showed that the hexane/isopropanol method is very efficient for extracting triacylglycerols, ceramides, and free fatty acids, and fairly efficient for extracting phosphatidylcholine. The same study indicated that different extraction solvents gave different results for different classes of lipids. Thus, the choice of the extraction method should take into consideration the targeted class of lipids, since no single method can cover them all. In addition, it should be adapted to the sample matrix. In our study, which was focused on plasma samples where triacylglycerols are the predominant class of lipids, the hexane/isopropanol was considered appropriate. We also considered the simplicity of the method and the fact that it is less time-consuming. We estimated that the time saved was approximately three minutes per sample as compared to the Bligh-Dyer method, a fact that counts especially when a large number of samples are prepared, such as in metabolomic assessments.

### 2.2. Simultaneous Quantification of 5 Fatty Acids

**Figure 1:**
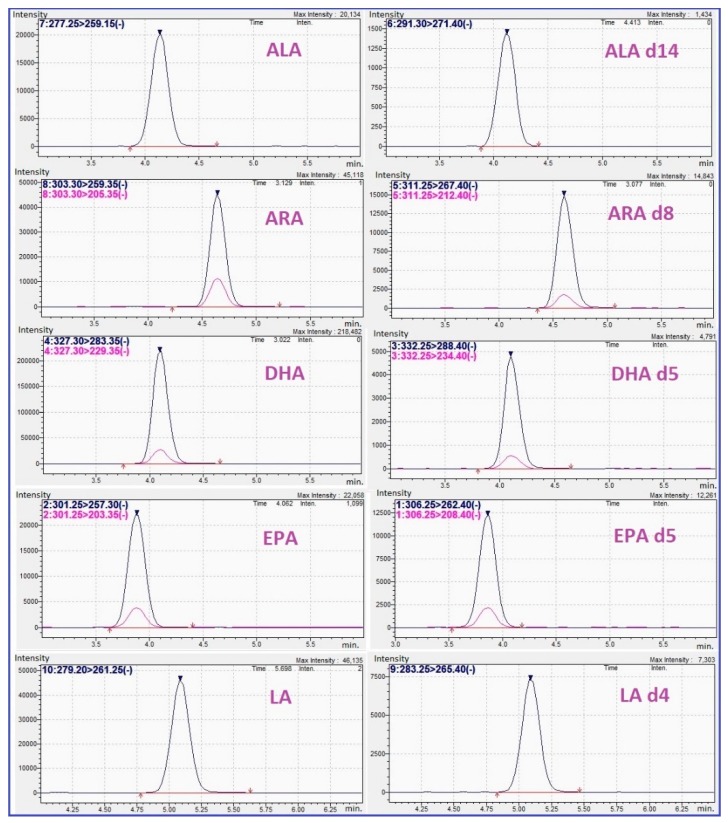
Multiple reaction monitoring (MRM) chromatograms of ALA, ALA-d14, ARA, ARA-d8, DHA, DHA-d5, EPA, EPA-d5, LA, and LA-d4 standards at concentration of 0.016, 0.2, 0.08, 0.1, 0.016, 0.1, 0.016, 0.1, 0.08, and 0.2 µg/mL respectively. Legend: ALA—α-linolenic acid, ARA—arachidonic acid, DHA—docosahexaenoic acid, EPA—eicosapentaenoic acid, LA—linoleic acid.

A multiple reaction monitoring (MRM) method was successfully developed for all selected FAs. Where possible, two transitions per compound were used. Thus, for ARA, DHA, and EPA we used 2 transitions while for ALA and LA only one transition was used. MRM chromatograms for each targeted FA are exemplified in Figure 1.

The limits of detection (LODs) were situated in a 0.8–10.7 nmol/L interval, while the limits of quantification (LOQs) were in a 2.4–285.3 nmol/L range (Table 1).

The LOQs were situated in low nanomolar range excepting for LA, for which the limit was in high nanomolar range but not considered an issue since its minimum observed level in samples was in micromolar range. Descriptive statistics on plasma FAs results are indicated in Table 2.

When using ammonium acetate in the mobile phase, previously used in other studies [21,22], the sensitivity was enhanced, probably due to the acetate anion improving negative ESI responses as indicated by Hua and Jenke [23].

Previous studies have shown that reverse phase C18 columns provided good separation for fatty acids [12,15]. In the HPLC optimization process, we also evaluated a high-pressure C18 column (Acquity UPLC 100 × 2.1 mm, 1.7 µm BEH C18 column, Waters, Milford, Massachusetts, USA), but the results were modest as no peak was detected for ALA at 0.016 µg/mL, and the peak intensities for the other targeted compounds were significantly lower (obtained MRM chromatograms are showed in Figure S3). Therefore, the Shim-Pack GIST-HP C18 was selected for further method development.

**Figure 2:**
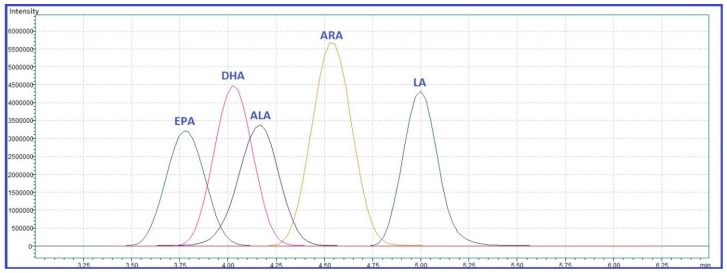
The overlaid total ion chromatograms of the targeted fatty acids. Data was obtained following analysis of a plasma sample (total FAs content). Legend: ALA—α-linolenic acid, ARA—arachidonic acid, DHA—docosahexaenoic acid, EPA—eicosapentaenoic acid, LA—linoleic acid.

When using the Shim-Pack GIST-HP C18 column with isocratic flow, elution started at 3.83 min with EPA, while the last was LA at 5.02 min (Figure 2). The HPLC method ended at 6.5 min. Even if the run was ended after less than 2 min after the last compound eluted, no carry-over was observed as no peaks were integrated from blank samples, even after running the highest concentrated standards (50 µg/mL). In addition, the RSD% of the retention times (Table 1) were low. The use of isocratic flow eliminated the need for column equilibration, allowing us to shorten the analytical cycle and to obtain a high sample throughput.

This LC-MS/MS method was designed considering the primary goal, which was to develop a method of quantification without the derivatization of fatty acids. Derivatization would have required several extra steps in an already time-consuming sample preparation protocol. Previously published methods using dimethylaminoethyl ester (DMAE) derivatives of fatty acids provided LODs in the low femtomoles range [12], however it required an additional incubation and extraction steps, adding more complexity to an already time-consuming protocol. Although derivatization provides unparalleled sensitivity, our method proved that FAs from human plasma can be reliably quantified without the need of derivatization.

In comparison with the GS-MS and GS-FID methods, which require an approximately 20 min analytical run [11,24], our method is significantly shorter, with a duration of only 6.5 min, which results in a massive time reduction after running a large number of samples.

### 2.3. Method Validation

All acceptance criteria were met. The calibration curves showed a very good correlation between concentration and response, R-squared for all calibrants was greater than 0.995, with no weighting method applied. All calibration curves were reproducible.

Accuracy was evaluated for four different concentrations for each standard. The RSD%s were all within the margin of ±15%. The highest RSD (9.1%) was found at the lowest calibrant for LA, a value that had a large margin inside the Food and Drug Administration (FDA) guide’s acceptance. Accuracy was also surveyed during each analytical run by inserting a quality control sample for every 10 samples, and allowance was set for the LabSolution software between 85% and 115%, as any deviation from this interval would invalidate the results. The RSD% for intra-assay and inter-assay precision test were also within the acceptable margin of 15%, demonstrating that the results are reproducible. The complete results for replicated analysis of standards (accuracy) and replicated analysis of samples (precision) are indicated in Table 3 and Table 4, respectively.

Recovery efficiency was >90% for all internal standards. Recovery reproducibility results for total fatty acids samples (RSDs) were: ALA-d14—5.2%, ARA-d8—3.3%, DHA-d5—11% EPA-d5—4.2% and LA-d4—10.1%; while for free fatty acids samples, the RSDs were: ALA-d14—7%, ARA-d8—9.3%, DHA-d5—4.6% EPA-d5—3.4% and LA-d4—7.6%. The obtained results showed that the response of internal standards was consistent and therefore the matrix effect was acceptable.

## 3. Materials and Methods

### 3.1. Participants and Samples

Two hundred obese children (97 males, 103 females) were recruited at the 2nd Paediatric Clinic of Clinical Emergency County Hospital Timisoara, Romania, in the context of a bigger study. Participants were aged 7–18 years, with BMI > +2 SD as compared to the World Health Organization (WHO) reference, and abdominal circumference above the 90th percentile. Their parents or other legal guardians, as well as the children themselves, were fully informed about the aims and methods of the study, and informed consent was obtained from legal guardians. The study was conducted in accordance with the Declaration of Helsinki, and was approved by the Ethics Committee of the “Victor Babes” University of Medicine and Pharmacy, Timisoara, Romania, and was registered at ClinicalTrials.gov (NCT02837367).

The blood samples were collected after overnight fasting using a 2 mL VACUTEST^®^ tube Sterile R interior, with K_3_EDTA 3.6 mg (Vacutest Kima srl, Arzegrande, Italy). Samples were then subjected to centrifugation at room temperature at 4000 rpm for 10 minutes. After centrifugation, the upper plasma was collected and stored at −70 °C until further use.

### 3.2. Standards and Reagents

Docosahexaenoic acid, Docosahexaenoic acid—d5, Eicosapentaenoic acid, Eicosapentaenoic acid—d5, Arachidonic acid, Arachidonic—d8, Linoleic acid, and α-Linolenic acid were purchased from Sigma-Aldrich (St. Louis, MO, USA). Linolenic Acid—d4 and α-Linolenic Acid—d14 were purchased from Cayman Chemical Company (Ann Arbor, MI, USA).

Methanol and Hexane were purchased from Honeywell (Seelze, Germany), potassium hydroxide from Chimreactiv (Bucharest, Romania), acetonitrile from AppliChem GmbH (Darmstadt, Germany), isopropanol, formic acid, and ammonium acetate from Merck (Darmstadt, Germany), and water from VWR International (Radnor, Pennsylvania, USA). All reagents were HPLC-grade.

### 3.3. Sample Preparation

#### 3.3.1. Lipid Extraction

Aliquots of 100 µL plasma were transferred to Eppendorf tubes and mixed with 10 µL of internal standard mixture containing 10 µg/mL DHA-d5, ARA-d8, and EPA-d5, and 10 µg/mL LA-d4 and ALA-d1. The lipids were then extracted with hexane/isopropanol, 3:2 *v*/*v* at a 1:10 sample/solvent ratio. The tubes were vortexed and maintained at −20 °C for 10 min, then centrifuged at 14,000 *g* at 4 °C for 5 min. The supernatant was collected, transferred to glass tubes, and dried under nitrogen flow. Then, 1 mL of 80% methanol was added, and the tubes were thoroughly mixed. Of this, a volume of 100 µL was transferred into an autosampler vial for free fatty acids analysis. The remaining aliquot was subjected to alkaline hydrolysis.

The Bligh–Dyer method [25] (Supplementary Material 1) was also used for comparison.

#### 3.3.2. Alkaline Hydrolysis

A volume of 100 µL of a solution of 0.3 M KOH in 80% methanol was added to the lipid extract. The mixture was incubated at 80 °C for 30 min. The tubes were allowed to cool, and then 10 µL of formic acid was added to neutralize the pH. For separating the fatty acids, 1 mL hexane was added, and the tubes were placed on a rotary mixer for 5 min. The tubes were briefly centrifuged at 1000× *g*, and then the top hexane layer was transferred into a clean glass tube and dried under nitrogen flow. The sediment was reconstituted in 1 mL of 80% methanol, of which 100 µL was transferred into an autosampler vial for analysis.

### 3.4. Preparation of Standard Solutions

Calibration standards were prepared in 80% methanol at concentrations of 0.08, 0.4, 2, 10 and 50 µg/mL for LA and ARA; 0.016, 0.08, 0.4, 2 and 10 µg/mL for DHA; and 0.0032, 0.016, 0.08, 0.4 and 2 µg/mL for EPA and ALA. Internal standards were added to all calibration standards at concentrations of 0.1 µg/mL for DHA-d5, ARA-d8, and EPA-d5, and of 0.2 µg/mL for LA-d4 and ALA-d14.

### 3.5. LC–MS/MS Analysis

The liquid chromatography–tandem mass spectrometry (LC-MS/MS) system comprised a Shimadzu UHPLC Nexera X2 system hyphenated to a triple quadrupole mass spectrometer LCMS-8045 (Shimadzu Corporation, Kyoto, Japan). The samples were kept in the autosampler (Nexera X2 SIL-30AC) at 5 °C. The chromatographic separation was performed using a 150 × 2.1 mm, 3 μm Shim-Pack GIST-HP C18 column equipped with a GIST-HP 10 × 1.5 mm, 3 μm guard column (Shimadzu Corporation, Kyoto, Japan). The LC system was composed of one Nexera X2 LC-30AD quaternary pump, a column oven (CTO-20AC) maintained at 40 °C, and a model DGU-20A5R degasser. The separation was performed using isocratic flow of a solvent composed of 90% acetonitrile, 10% water, and 2 mM ammonium acetate. The flow rate was set at 0.21 mL/min. The injection volume used was 10 μL.

The mass spectrometer was operated with an electrospray ionization (ESI) source in negative mode. The interface temperature was set 300 °C while the desolvation line and heat block temperatures were set at 250 and 400 °C, respectively. The interface voltage was maintained at 3kV. Nitrogen was used as nebulizing gas at a flow of 3 L/min, while drying gas and heating gas flows (also nitrogen) were maintained at 10 L/min. Argon was used as collision gas at 230 kPa.

The MS/MS parameters (Table 5) were optimized by direct injection of 1 μL volumes of standards at 100 μg/mL.

The MS data were processed using LabSolution software (v5.91/2017, Shimadzu Corporation, Kyoto, Japan). Spectra was smoothed using a standard method with a width of 20 s. Peak integration was performed using the i-PeakFinder algorithm with a 1-degree baseline following. Peak identification was based on absolute retention time (RT) with a window of 2%. No reference RT update was used. Reference ion ratio allowance (for ALA, ALA-D14, DHA, DHA-d5, EPA, and EPA-d5) was set to 50%. The quantitation method was based on 5-point calibration and using peak areas. Calibration curves were set to linear with no forcing or weighting applied.

### 3.6. Method Validation

Method validation procedures followed the recommendations and the acceptance criteria found in the FDA guide for bioanalytical method validation [26] addressing calibration curve, accuracy, precision, recovery, quality control samples, and sensitivity.

Calibration curves were obtained using the LabSolution software (v5.91/2017, Shimadzu Corporation, Kyoto, Japan) where the acceptance criterion for calibration points was ±20% of the theoretical concentrations.

For evaluating accuracy, 10 replicate analyses of standards were performed at the following concentrations: 0.08, 0.4, 2, 10 µg/mL for ALA, DHA, LA, and ARA, and 0.016, 0.08, 0.4, 2 µg/mL for EPA. Relative standard deviation (RSD%) was calculated using analyte peak area. Also, the RSD was calculated for the retention times. The acceptance criterion was ±15% RSD.

For evaluating precision, three plasma samples were pooled, extracted, and analysed 10 times in the same day (within-run precision) and five times in different days (between-run precision). RSD was calculated for the obtained results (concentration), and the acceptance criterion was ±15%.

For recovery, both efficiency and reproducibility were evaluated. The efficiency was calculated by comparing the internal standard peak areas of standards and real samples. Reproducibility was evaluated by calculating the RSD of the internal standard peak area from 20 samples. The FDA guide does not mention acceptance criterion in this case, however it recommends that internal standard recovery should be consistent and reproducible. Therefore, we set the acceptance criterion to ±15%.

One quality control (QC) sample and one blank (consisting of 80% methanol) were analysed for every 10 samples. The allowance range was set at 85–115%.

For sensitivity evaluation, the limit of detection (LOD) and limit of quantification (LOQ) were automatically calculated by the LabSolution software (v5.91/2017, Shimadzu Corporation, Kyoto, Japan). LOD was established as the lowest concentration of the calibration standard detected with a signal-to-noise (S/N) ratio ≥ 3:1 while the LOQ was established as the lowest concentration of the calibration standard detected with a S/N ratio ≥ 10:1.

### 3.7. Statistics

RSD% used in validation was calculated with LabSolution software (v5.91/2017, Shimadzu Corporation, Kyoto, Japan).

Descriptive statistics were performed using Minitab version 17.1.0 (Minitab, Inc. State College, PA, USA).

## 4. Conclusions

The developed and validated method described offers a simplified extraction procedure without the need of derivatization, and a fast and reproducible LC-MS/MS quantification. It is suitable for quantifying *n*-3 and *n*-6 fatty acids in human plasma, in both free and total forms.

Due to the fact the derivatization is not needed, the sample preparation is less time-consuming. The LC-MS/MS proved significantly faster than previously published GS-MS methods. Combining these two factors, the proposed workflow is significantly less time-consuming than existing GS–MS and LC-MS methods.

The method can be used to assess changes in fatty acid metabolism, which have implications in obesity, type 2 diabetes, insulin resistance, and interrelationship with other metabolic pathways.

## Figures and Tables

**Table 1:** Retention times (RT) and their relative standard deviation (RSD), limit of detection (LOD), and limits of quantification (LOQ) for each compound.

Compound	RT (min)	RSD% for RT	LOD (nmol/L)	LOQ (nmol/L)
ALA	4.17	1.10	3.59	17.96
ARA	4.60	0.50	3.28	13.14
DHA	4.08	0.60	0.82	2.47
EPA	3.83	0.50	2.02	6.12
LA	5.07	0.50	10.70	285.26
ALA D14	4.07	0.50	N/A	N/A
ARA D8	4.53	0.33	N/A	N/A
DHA D5	4.05	0.55	N/A	N/A
EPA D5	3.81	0.50	N/A	N/A
LA D4	5.02	0.35	N/A	N/A

Legend: RT—retention time, RSD—relative standard deviation, LOD—limit of detection, LOQ—limit of quantification, ALA—α-linolenic acid, ARA—arachidonic acid, DHA—docosahexaenoic acid, EPA—eicosapentaenoic acid, LA—linoleic acid.

**Table 2:** Descriptive statistics of quantification results: Minimum, median, and maximum concentrations found, and the standard deviation for each quantified fatty acid (total and free).

Compound		Min. Conc. (µmol/L)	Mean Conc. (µmol/L)	Max. Conc. (µmol/L)	StDev (µmol/L)
ALA	total	8.99	20.14	41.49	9.80
	free	0.50	6.36	23.16	4.10
ARA	total	215.40	387.10	693.10	119.70
	free	3.18	8.36	32.25	4.50
DHA	total	131.20	297.40	544.20	132.30
	free	1.50	8.40	42.36	5.70
EPA	total	2.14	9.14	22.02	5.57
	free	0.09	0.32	2.90	0.28
LA	total	660.40	1012.00	1555.50	247.20
	free	25.60	191.50	601.10	97.05

Legend: StDev—standard deviation, ALA—α-linolenic acid, ARA—arachidonic acid, DHA—docosahexaenoic acid, EPA—eicosapentaenoic acid, LA—linoleic acid.

**Table 3:** The results for replicated analysis of standards (test accuracy). Each standard was analysed 10 times and the peak area was used to calculate the relative standard deviation (RSD).

Compound	RSD %					Mean RSD%
	0.016 µg/mL	0.08 µg/mL	0.4 µg/mL	2 µg/mL	10 µg/mL	
ALA	N/A	6.9	2.7	5.2	4.7	4.9
ARA	N/A	8.6	7.39	2.7	6	6.2
DHA	N/A	3.3	7	2.6	6.1	4.7
EPA	6.9	2.3	6.1	6.2	N/A	5.4
LA	N/A	7.4	2.7	6	6.7	5.7

Legend: ALA—α-linolenic acid, ARA—arachidonic acid, DHA—docosahexaenoic acid, EPA—eicosapentaenoic acid, LA—linoleic acid.

**Table 4:** The results for replicated analysis of samples (test precision). The sample was analysed 10 times in the same day (within-run precision) and 5 times in different days (between-run precision). The obtained concentrations were used to calculate the relative standard deviation (RSD%).

Compound		Intra-Assay RSD%	Inter-Assay RSD%
ALA	total	8.05	12.60
	free	7.81	13.10
ARA	total	1.92	6.50
	free	2.01	7.10
DHA	total	5.54	7.45
	free	5.20	7.70
EPA	total	3.00	8.10
	free	3.40	9.40
LA	total	2.89	6.05
	free	3.34	7.60

Legend: ALA—α-linolenic acid, ARA—arachidonic acid, DHA—docosahexaenoic acid, EPA—eicosapentaenoic acid, LA—linoleic acid.

**Table 5:** Optimized MRM transitions.

Compound	Transition (*m*/*z*)	Dwell Time (ms)	Q1 Pre-Bias (V)	Collision Energy	Q3 Pre-Bias (V)
ALA	277.25→259.15	100	10	16	16
ALA D14	291.30→271.40	100	14	18	12
DHA	327.30→283.35 327.30→229.35	100 100	12 12	10 12	18 15
DHA D5	332.25→288.40 332.25→234.40	100 100	17 17	11 12	12 14
LA	279.20→261.25	100	11	18	27
LA D4	283.25→265.40	100	14	18	26
ARA	303.30→259.35 303.30→205.35	100 100	15 15	12 13	16 12
ARA D8	311.25→267.40 311.25→212.40	100 100	16 16	13 13	12 13
EPA	301.25→257.30 301.25→203.30	100 100	11 16	10 12	11 12
EPA D5	306.25→264.40 306.25→208.40	100 100	11 15	11 12	11 12

## Supplementary Materials

The Supplementary Materials are available online.
