# Supplementary material for: Development and Validation of a LC–MS/MS-Based Assay for Quantification of Free and Total Omega 3 and 6 Fatty Acids from Human Plasma
Source: Molecules. 2019 Jan 20;24(2):360. doi: 10.3390/molecules24020360 (PMC6359656; doi:10.3390/molecules24020360)
Supplement: Supplementary file 1 [file molecules-24-00360-s001.zip › supple-molecules-422910/Supplementary material 1.pdf]

Supplementary material 1

Bligh-Dyer lipid extraction method

The methodology was adapted from Bligh-Dyer method [18]. Aliquots of 100 μL plasma were transferred in Eppendorf tubes and mixed with 10 μL of internal standard mixture containing 10 μg/mL DHA-d5, ARA-d8 and or EPA-d5, and 10 μg/mL LA-d4 and ALA-d1. 750 μL of chloroform/methanol mixture (1:2, v/v). The tubes were vortexed and maintained at -20°C for 10 min, then centrifuged at 14,000 *g* at 4°C for 5 min. The supernatant was transferred into a clean tube. 250 μL of chloroform and 250 μL water were added. The tubes were placed on a rotary mixer for 5 min and then centrifuged for 5 min at 1000 g.

The bottom layer was collected, transferred in a glass tubes and dried under nitrogen flow. 1 mL of 80% methanol was added, and the tubes were thoroughly mixed. Of this, a volume of 100 μL was transferred into an autosampler vial for free fatty acids analysis. The remaining aliquot was subjected to alkaline hydrolysis as described in section 3.3.2.
