# Supplementary material for: Development and Validation of a LC–MS/MS-Based Assay for Quantification of Free and Total Omega 3 and 6 Fatty Acids from Human Plasma
Source: Molecules. 2019 Jan 20;24(2):360. doi: 10.3390/molecules24020360 (PMC6359656; doi:10.3390/molecules24020360)
Supplement: Supplementary file 1 [file molecules-24-00360-s001.zip › supple-molecules-422910/Supplementary material 2.pdf]

Supplementary material 2

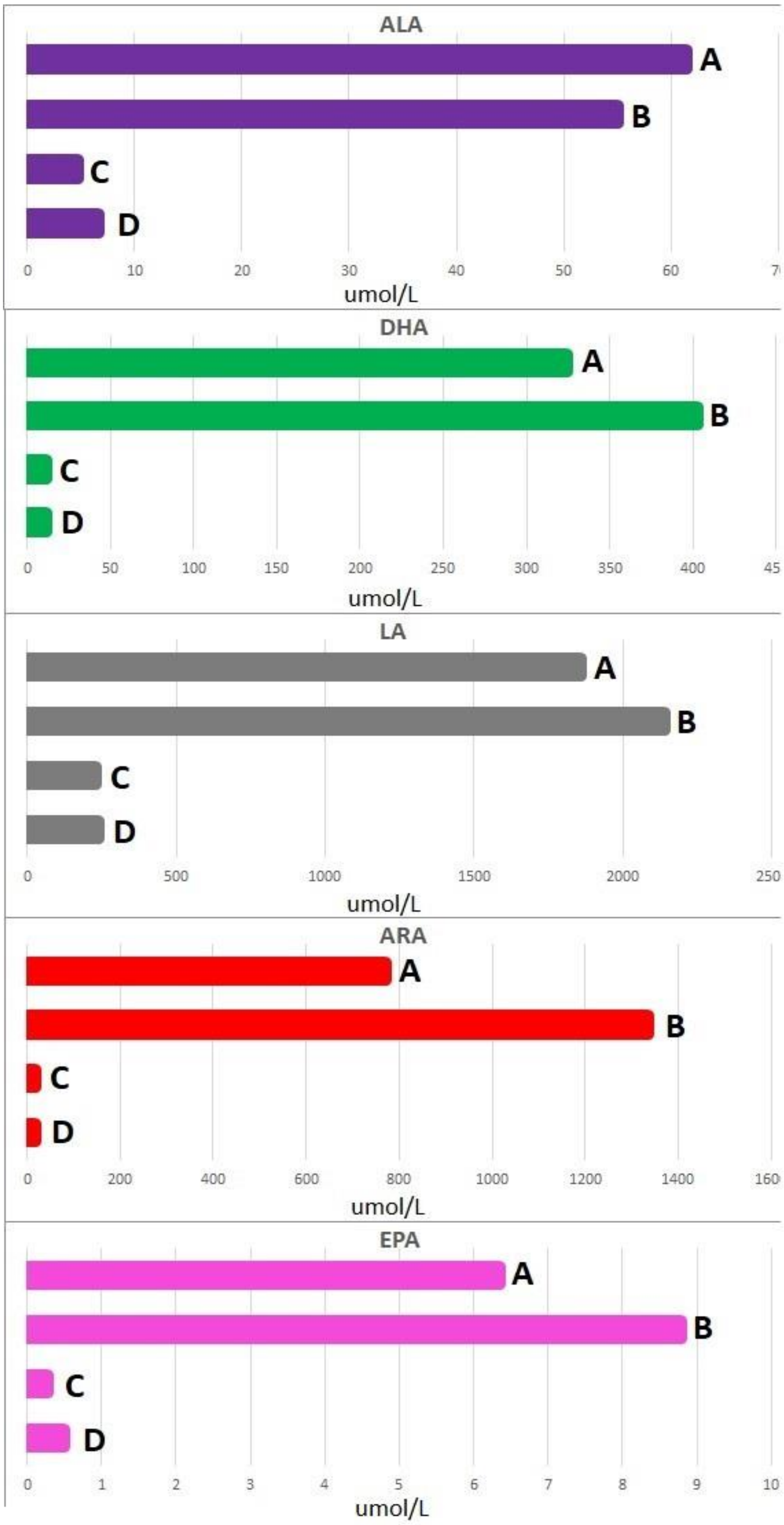

Figure S2. Evaluation of extraction protocol. Graphs show the levels of each FA in both free and total forms extracted with Bligh/Dyer method and hexane/isopropanol: A – FA from total lipids extracted using Bligh/Dyer method, B - FA from total lipids extracted with hexane/isopropanol, C – FA in free form extracted using Bligh/Dyer method, D – FA in free form extracted with hexane/isopropanol.

Legend: ALA—α-linolenic acid, ARA—arachidonic acid, DHA—docosahexaenoic acid, EPA—eicosapentaenoic acid, LA—linoleic acid
