# Supplementary material for: Development and Validation of a LC–MS/MS-Based Assay for Quantification of Free and Total Omega 3 and 6 Fatty Acids from Human Plasma
Source: Molecules. 2019 Jan 20;24(2):360. doi: 10.3390/molecules24020360 (PMC6359656; doi:10.3390/molecules24020360)
Supplement: Supplementary file 1 [file molecules-24-00360-s001.zip › supple-molecules-422910/Supplementary material 3.pdf]

Suplementary material 3

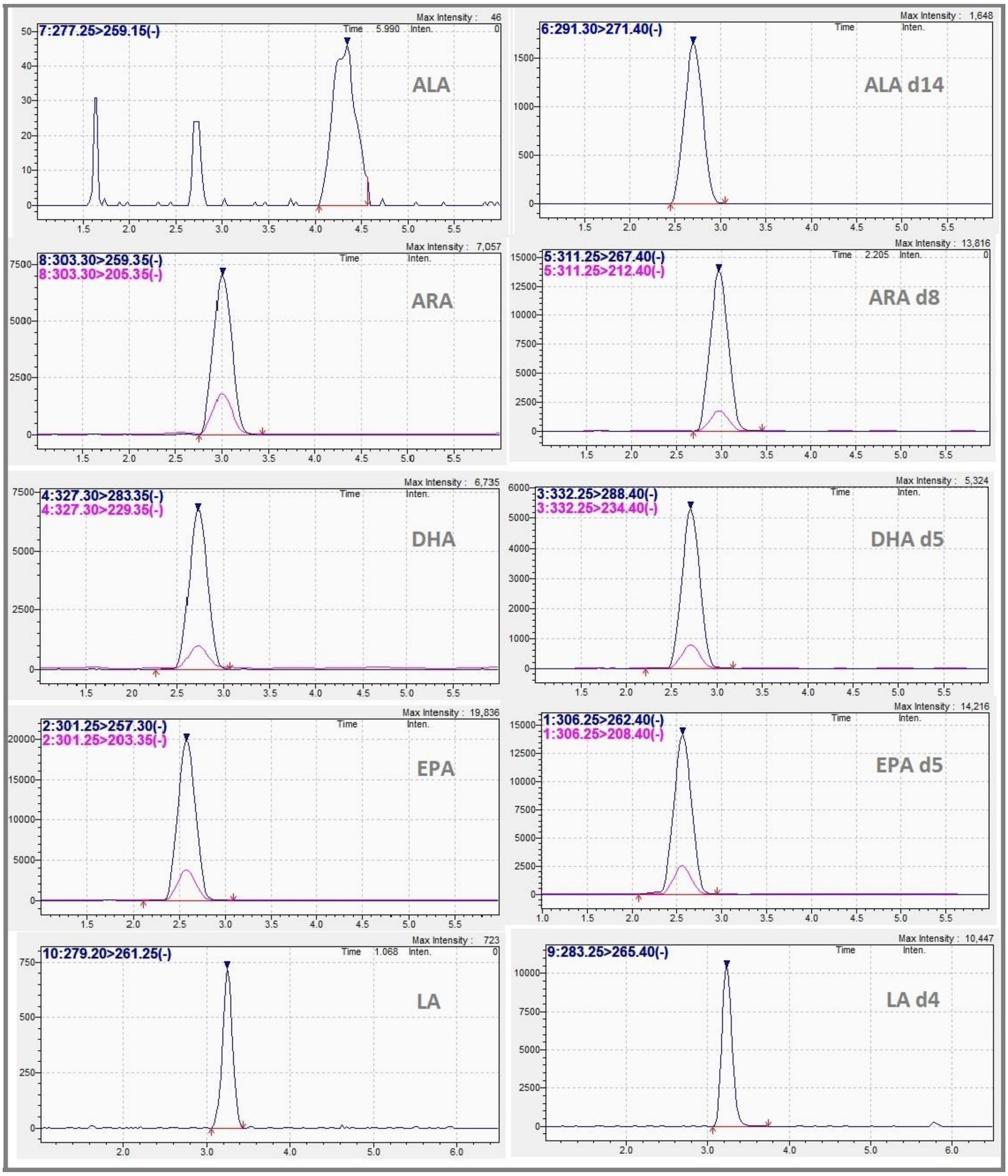

Figure S3. Evaluation of Acquity UPLC BEH C18 column. MRM chromatograms of ALA, ALA-d14, ARA, ARA-d8, DHA, DHA-d5, EPA, EPA-d5, LA and LA-d4 standards at concentration of 0.016, 0.2, 0.08, 0.1, 0.016, 0.1, 0.016, 0.1, 0.08 and 0.2 μg/mL respectively.

Legend: ALA—α-linolenic acid, ARA—arachidonic acid, DHA—docosahexaenoic acid, EPA—eicosapentaenoic acid, LA—linoleic acid
